# Supplementary material for: Cytokine-Induced Guanylate Binding Protein 1 (GBP1) Release from Human Ovarian Cancer Cells
Source: Cancers (Basel). 2020 Feb 19;12(2):488. doi: 10.3390/cancers12020488 (PMC7072386; doi:10.3390/cancers12020488)
Supplement: Supplementary file 1 [file cancers-12-00488-s001.zip › cancers-702123-suppl-final version/cancers-702123-suppl-figures-final.docx]

Supplementary Material: Cytokine-Induced Guanylate Binding Protein 1 (GBP1) Release from Human Ovarian Cancer Cells

Grazia Carbotti, Andrea Petretto, Elisabeth Naschberger, Michael Stürzl, Stefania Martini, Maria Cristina Mingari, Gilberto Filaci, Silvano Ferrini and Marina Fabbi





**Figure S1.** The volcano plot illustrates the results of secretome data obtained from the different IL-27 and IFN-γ stimuli respect to the control. The proteomes are compared starting from a value of FDR <0.05 and S0> 0.1. Black dots are the proteins that exceed the acceptability threshold. GBP1 protein, statistically significant, is indicated in both treatments.

**
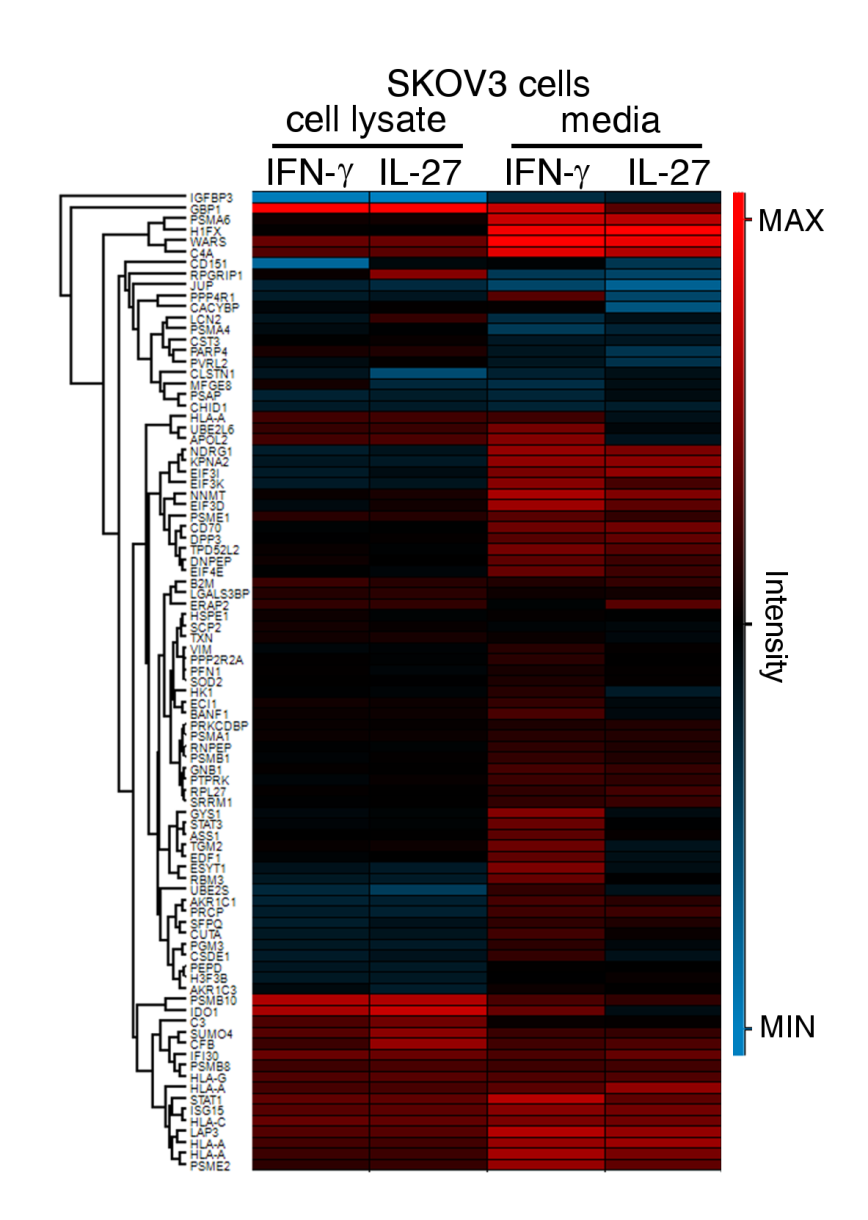

Figure S2.** Fold change heatmap of the 89 proteins common to the two experiments, cell lysate and conditioned media, selected from the Venn diagram and plotted using Perseus software.


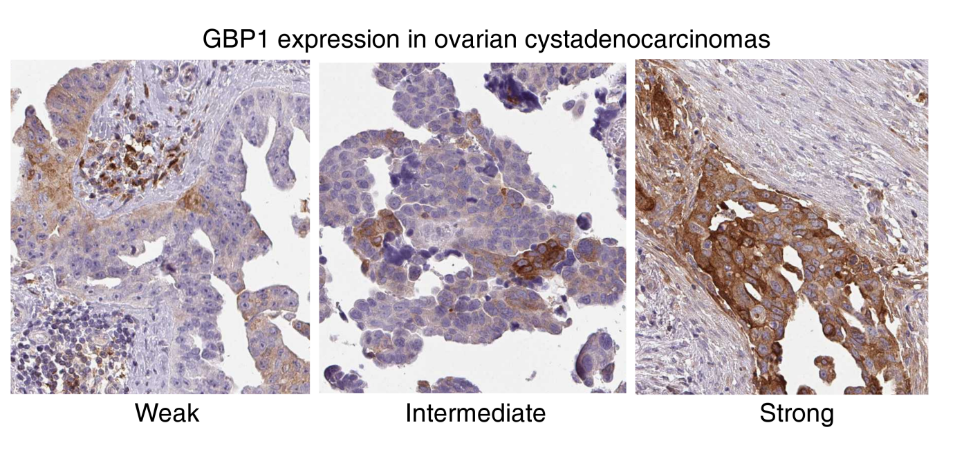


**Figure S3.** Immunohistochemistry confirmed the presence of GBP1 in the neoplastic EOC tissues (data from the Human Protein Atlas: https://www.proteinatlas.org/).





**Figure S4.** Western blot analysis of tumor cell-enriched fractions from EOC ascites shows constitutive GBP1 expression and STAT1 and STAT3 tyrosine phosphorylation. * indicates lanes from a blot re-probed for GBP1, where phosphorylated STAT3, STAT1 and actin were presented in previous articles (supplementary figure 4 of reference 10).





**Figure S5.** Kaplan-Meier analysis of patients survival in a subset of 14 EOC patients with available follow-up, stratified on the median value of soluble GBP1 as detected by ELISA in ascites fluids. P value not significant.





**Figure S6.** Soluble GBP1 is present in the liquid phase but not in the vesicular fractions of ascites A32. **A, B:** Ascites were fractionated by centrifugation at 3,000, 10,000 or 100,000× *g*. Anti-GBP1 immunoprecipitated molecules (IP GBP1) from the supernatant fractions or the corresponding micro-vesicle fractions were analyzed by Western blot, which showed the 67 kDa GBP1 (arrow) only in the supernatants. **C**: Extracellular Microvesicles preparations from ascites contain the exosome marker ALIX (<http://exocarta.ludwig.edu.au>) mainly in the 100,000× *g* fractions.


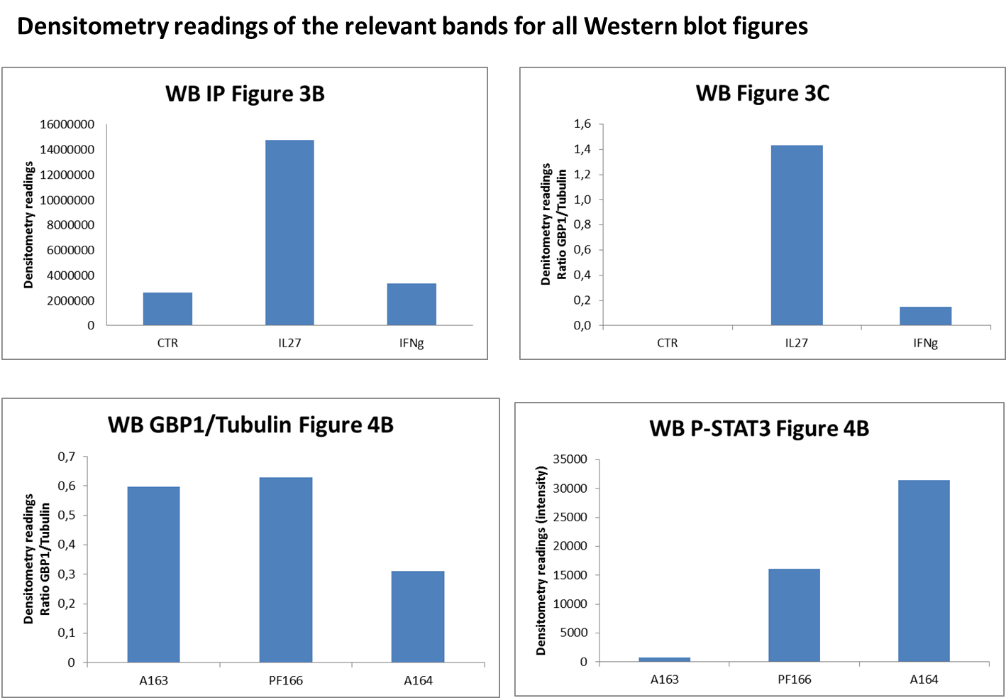


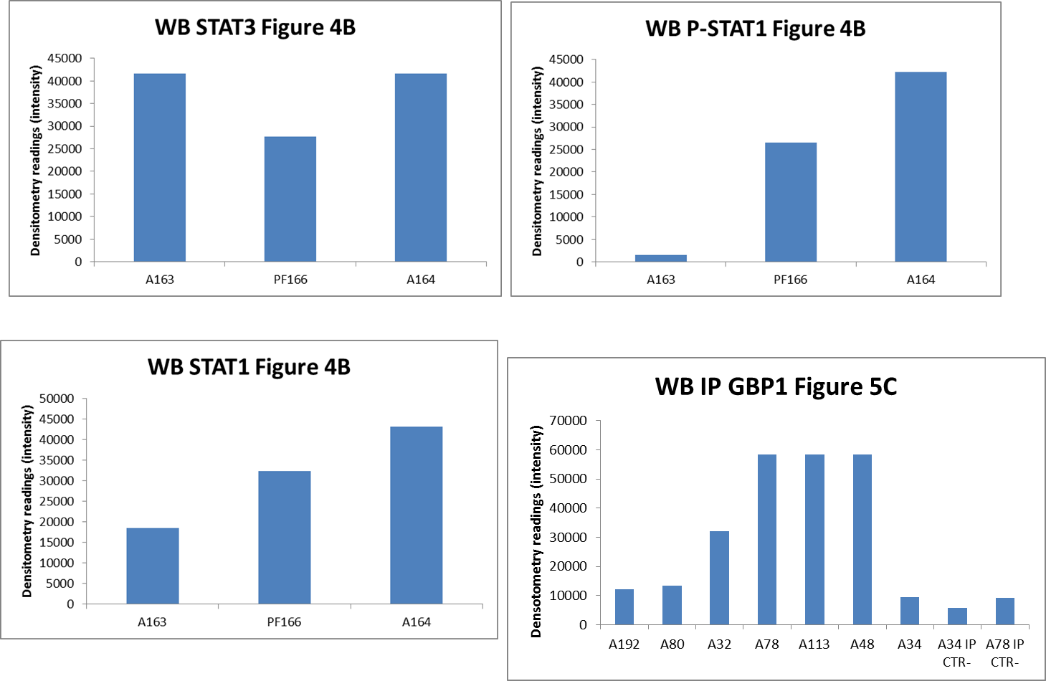


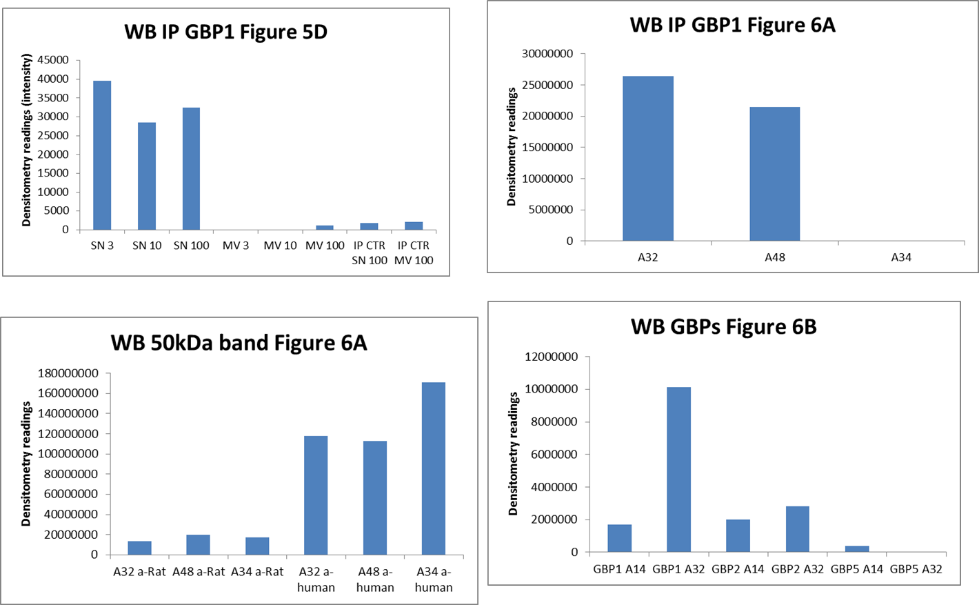


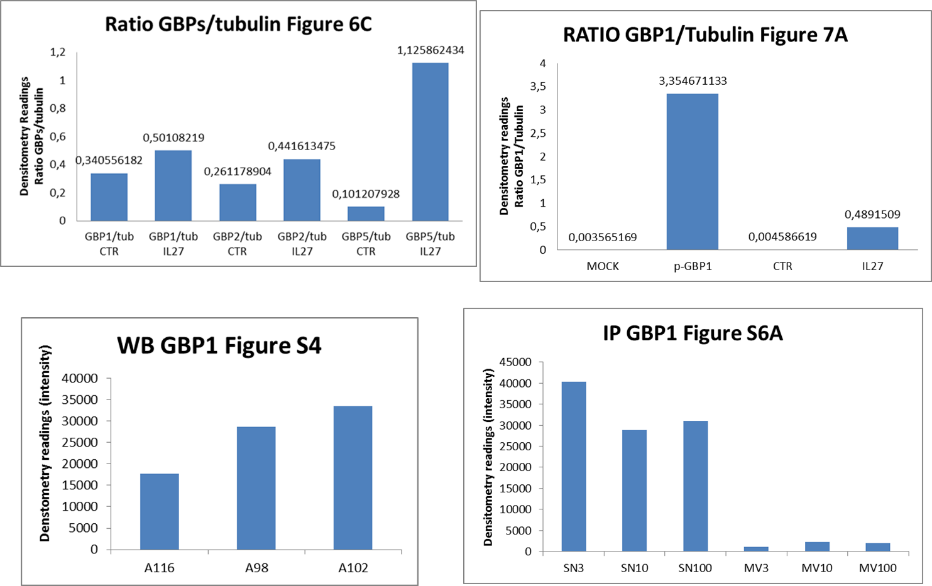


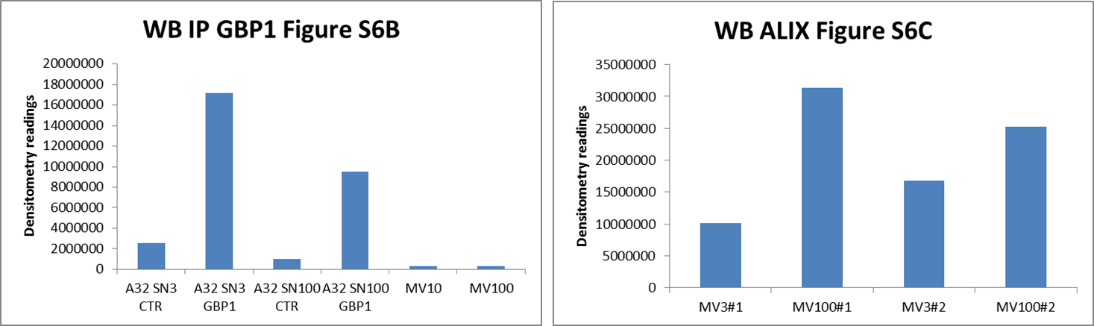


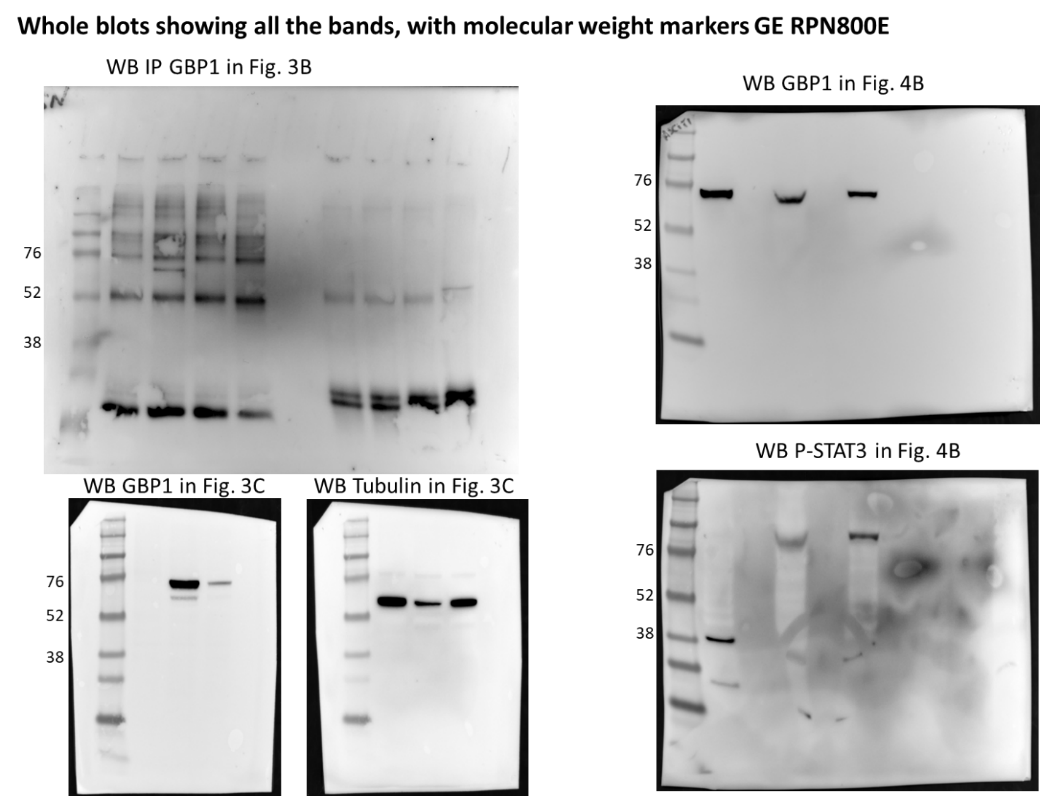


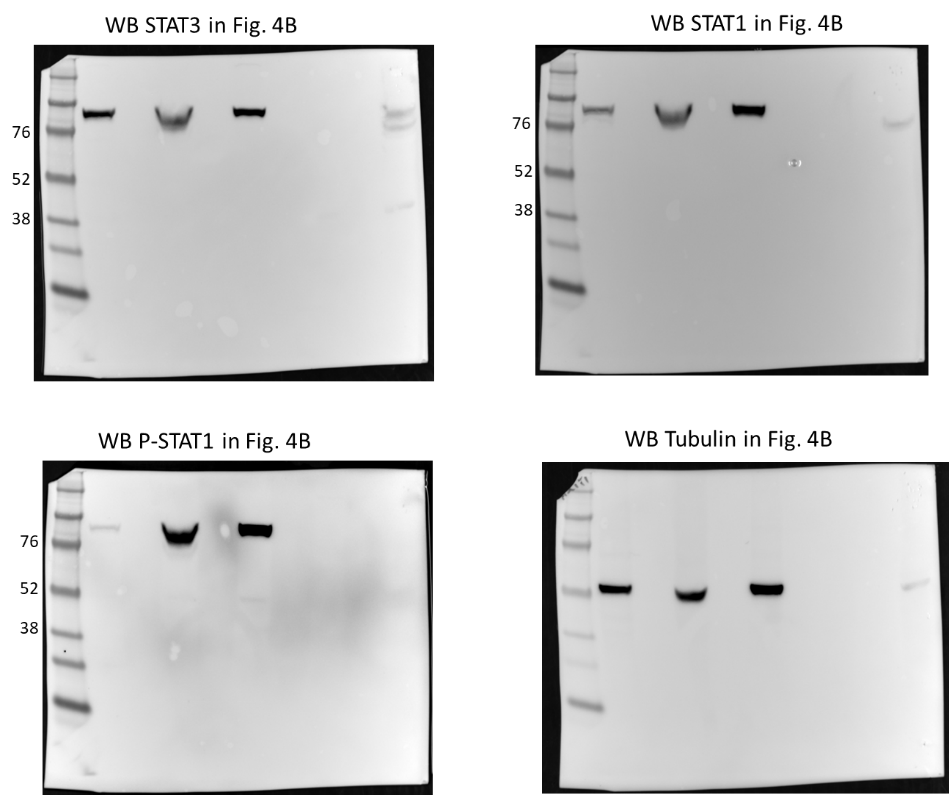


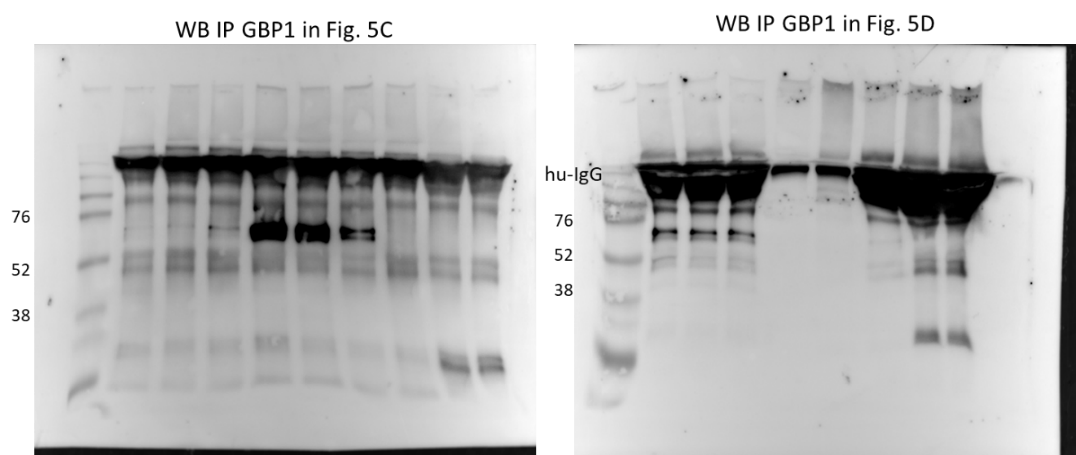


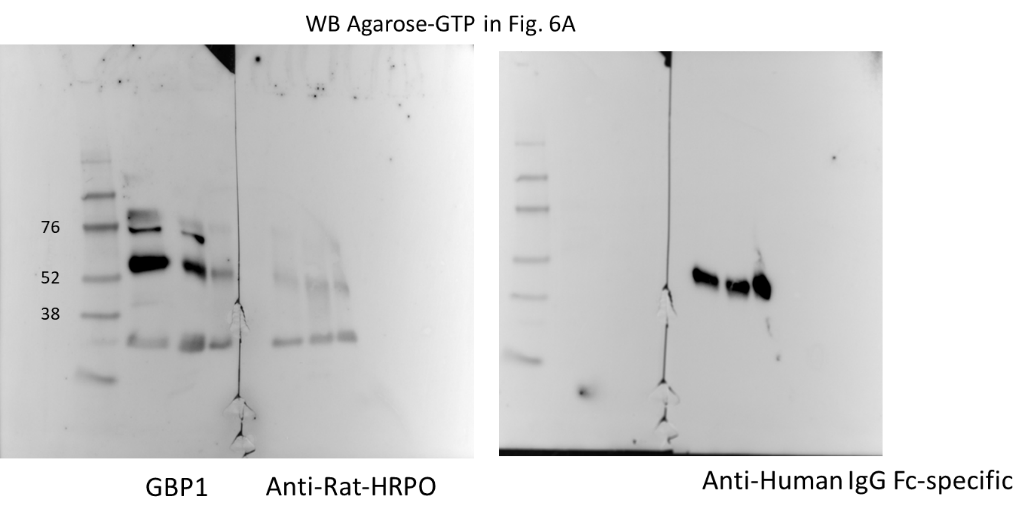


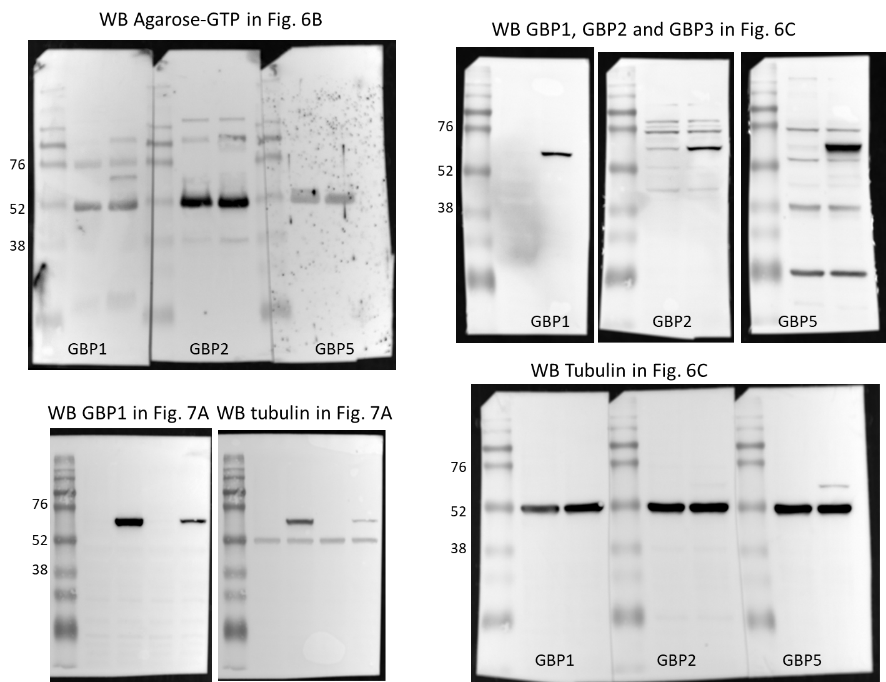


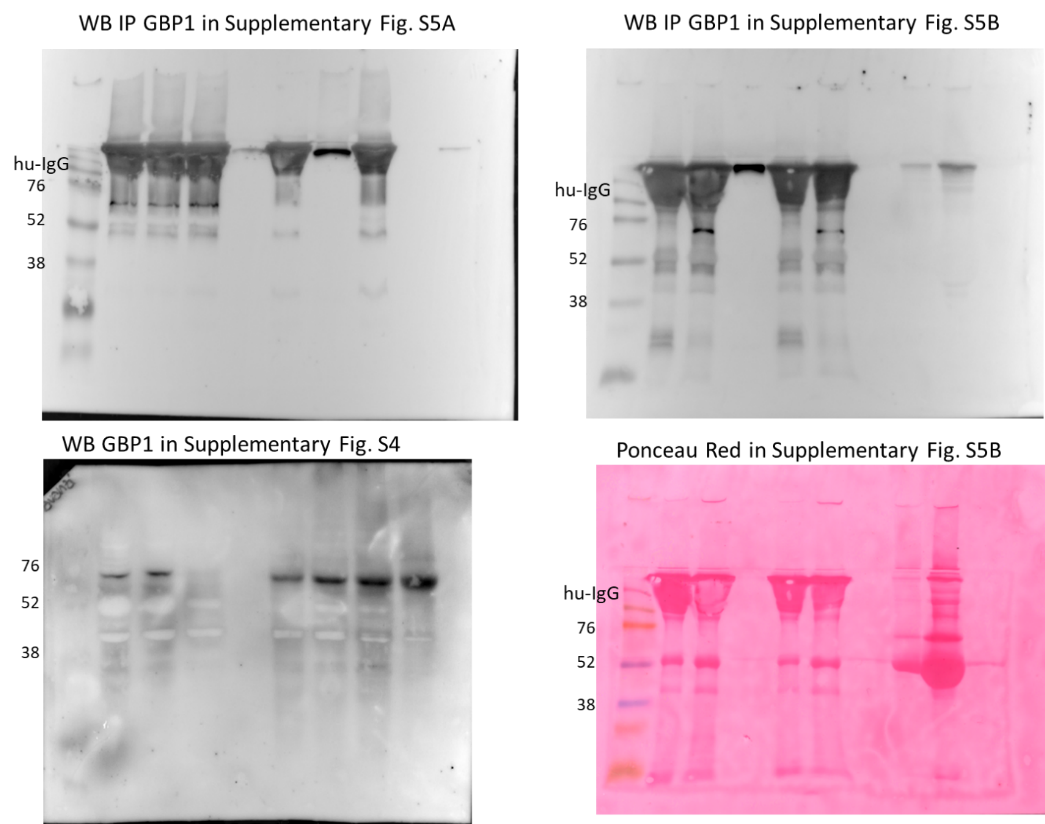


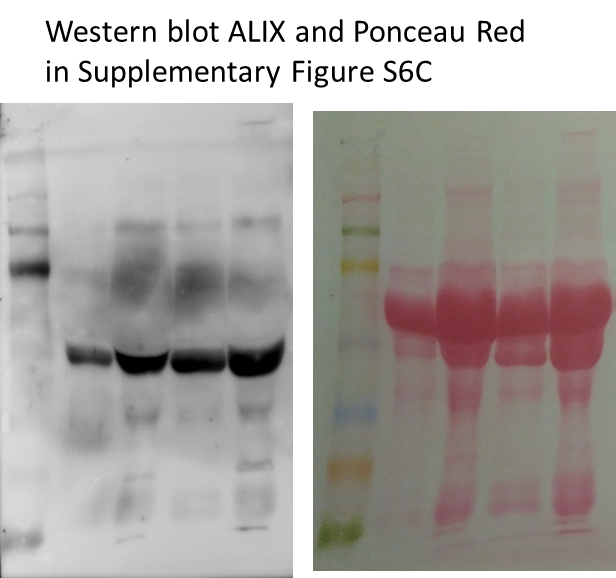


**Figure 7.** Densitometry readings or intensity ratio of the relevant bands for all Western blot figures. Whole blots showing all the bands with all molecular weight markers (GE RPN 800E) are shown for each figure panel.

**Table S6.** Distribution by tumor characteristics for EOC patients with evidence of disease.

| Characteristics | *N.* of cases (%)  ASCITES^1^ | *N.* of cases  SERA | *N.* of cases  PAIRED^2^ |
| --- | --- | --- | --- |
| Total | 30 (100) | 26 (100) | 20 (100) |
| Age |  |  |  |
| >55 years | 21 (70) | 20 (77) | 16 (80) |
| ≤55 years | 5 (17) | 4 (15) | 2 (10) |
| NA^3^ | 4 (13) | 2 (8) | 2 (10) |
| Stage^4^ |  |  |  |
| I | 3 (10) | 4 (15) | 2 (10) |
| II | 0 (0) | 1 (4) | 0 (0) |
| III | 16 (53) | 14 (54) | 12 (60) |
| IV | 6 (20) | 4 (15) | 3 (15) |
| NA | 5 (17) | 3 (12) | 3 (15) |
| Histotype |  |  |  |
| Serous | 17 (57) | 18 (69) | 13 (65) |
| Endometrioid | 2 (7) | 3 (12) | 2 (10) |
| Mucinous | 1 (3) | 0 (0) | 0 (0) |
| Others | 3 (10) | 1 (4) | 1 (5) |
| NA | 7 (23) | 4 (15) | 4 (20) |
| Grade |  |  |  |
| 1 | 0 (0) | 2 (8) | 0 (0) |
| 2 | 7 (23) | 10 (38) | 7 (35) |
| 3 | 13 (43) | 10 (38) | 9 (45) |
| Borderline | 2 (7) | 1 (4) | 1 (5) |
| NA | 8 (27) | 3 (12) | 3 (15) |

^1^ Peritoneal fluid (intra-surgery) or ascites ^2^ Paired serum and peritoneal fluid or ascites sample ^3^ NA not available ^4^ stage at sampling.

| 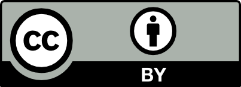 | © 2020 by the authors. Licensee MDPI, Basel, Switzerland. This article is an open access article distributed under the terms and conditions of the Creative Commons Attribution (CC BY) license (http://creativecommons.org/licenses/by/4.0/). |
| --- | --- |
